# The Improved DC Breakdown Strength Induced by Enhanced Interaction between SiO_2_ Nanoparticles and LLDPE Matrix

**DOI:** 10.3390/molecules28134908

**Published:** 2023-06-22

**Authors:** Yaqing Lu, Yuyao Liu, Yujie Tong, Huili Cheng, Di Yang, Jiandong Ding, Qiyang Guo

**Affiliations:** School of Chemistry and Chemical Engineering, Nantong University, Nantong 226019, China

**Keywords:** LLDPE, nanocomposites, SiO_2_, grafting onto, DC breakdown strength

## Abstract

Direct current (DC) power transmission systems have received great attention because it can easily integrate many types of renewable energies and have low energy loss in long-distance and large-capacity power transmission for electricity global sharing. Nanoparticles (NPs) have a positive effect on the insulation properties of polymers, but weak interaction between NPs and polymer matrix greatly decreases the effort of NPs on the enhancement of insulation properties, and thereby limits its engineering application. In this work, grafting strategy was used to link the modified NPs and polymer matrix to improve their interactions. Silica NPs (SiO_2_-NPs) were modified by 3-(methacrylyloxy) propyl-trimethoxysilane (MPS) to introduce highly active groups on the SiO_2_-NPs surface, followed by the pre-irradiated linear low-density polyethylene (LLDPE) being easily grafted onto the MPS modified SiO_2_-NPs (MPS-SiO_2_-NPs) in the melt blending process to obtain LLDPE-g-MPS-SiO_2_-NPs nanocomposites. Fourier-transform infrared (FT-IR) spectrum and X-ray photoelectron spectroscopy (XPS) confirm the successful incorporation of MPS into SiO_2_-NPs. Transmission electron microscopy (TEM) verifies that the modified SiO_2_-NPs exhibits more uniform distribution. The rheology result shows that the interaction between MPS-SiO_2_-NPs and LLDPE significantly improves. More importantly, the LLDPE-g-MPS-SiO_2_-NPs nanocomposites displays superior DC breakdown strength to that fabricated by conventional modification methods. When the addition of MPS-SiO_2_-NPs is 0.1 wt%, the highest DC breakdown strength values of 525 kV/mm and 372 kV/mm are obtained at 30 °C and 70 °C, respectively, and high DC breakdown strength can be well maintained in a wide loading range of NPs.

## 1. Introduction

The requirements of electric energy have extensively increased with the development of modern society. Traditional power generation has caused large carbon emission that contributes to severe environment issues. The wide spread of carbon neutrality has inspired the development of renewable energy sources, such as wind energy and solar energy [1]. Among them, direct current (DC) power transmission systems have received great attention in the past decade because they can easily integrate these types of renewable energies and have low energy loss in the long-distance and large-capacity power transmission for electricity global sharing [2]. An effective and common strategy to improve power capacity is to increase the applied voltage level of the high voltage DC cables. Because the dielectric breakdown of polymeric insulating materials is responsible for power cable failures in most cases due to over-voltage, internal defects, over-temperature and other elements [3], polymeric insulating materials with high DC breakdown strength is of great significance in the operation stability of power transmission system.

Recently, voltage stabilizers with high electron affinity were used to trap high energy electrons to increase the DC breakdown strength of polymers [4,5,6], while the voltage stabilizers are incompatible with polymer matrix and easily migrating. Englund et al. found that grafting alkyl side chains onto voltage stabilizers could enhance the solubility of voltage stabilizers [7,8,9,10]. To fundamentally inhibit the migration of low-molecule voltage stabilizer, grafting the voltage stabilizers onto polymer chains has also been studied. Yao and co-workers grafted the 4-Acryloxy Acetophenone onto LLDPE by reaction blending, which maintained the DC breakdown strength over 400 kV/mm during a long research period at high temperature [11]. Li et al. grafted 4-allyloxy-2-hydroxybenzophenone (AOHBP) onto low density polyethylene (LDPE) chains during peroxide cross-linking process, which increased the electrical tree initiation voltage of cross-linked polyethylene (XLPE) [12].

Nanoparticles have been widely concerned because of their great role in improving the optical, magnetic, electrical properties of materials [13,14,15]. Doping a small number of nanoparticles (NPs), such as ZnO [16], Al_2_O_3_ [17], MgO [18], TiO_2_ [19] and SiO_2_ [20], into polymer matrix can largely improve DC breakdown strength of polymers. However, such NPs that incorporated into the polymeric matrixes are easily agglomerated due to their high surface energy, leading to a troublesome fabrication or stabilization of uniform dispersed NPs [21,22]. Therefore, extensive efforts have been made on improving the compatibility between NPs and polymer matrix to form excellent interface structure [23]. Surface modification strategy is a simple but effective way to improve the compatibility between polymer matrix and NPs for preferably uniform dispersion [24,25]. Gao et al. grafted the voltage stabilizer onto the NPs which can not only enhance the uniform dispersion of NPs in the polymeric matrixes, but also efficiently inhibit the migration of voltage stabilizer [26]. Grafting polymers onto NPs has also been studied. For example, poly(stearyl methacrylate) was been grafted onto SiO_2_-NPs to enhance DC breakdown strength and reduce space charge accumulation [27]. Yao et al. grafted polystyrene onto both polymer matrix chains and NPs surface, so the polystyrene plays role as a bridge to link polymer matrix and NPs [28]. Meanwhile, using “grafting to” strategy, EPDM was been grafted onto ZnO NPs to improve insulating properties of XLPE [29].

**Scheme 1:**
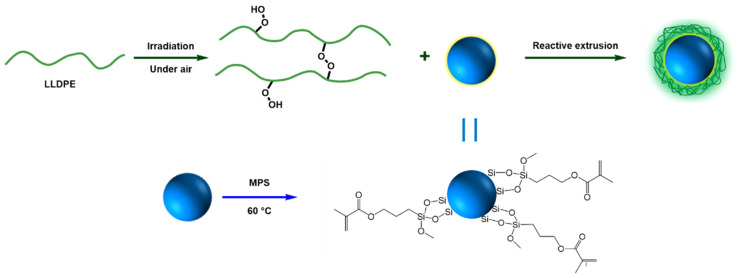
Schematic illustration of the LLDPE grafted with SiO_2_-NPs.

In this article, in order to investigate the influence of interaction between polymer matrix and NPs on DC breakdown strength, the SiO_2_ nanoparticles (SiO_2_-NPs) were first surface modified by 3-(methacrylyloxy) propyl-trimethoxysilane (MPS), forming MPS modified SiO_2_-NPs (MPS-SiO_2_-NPs), to introduce the highly active functional groups on SiO_2_-NPs. Then, linear low-density polyethylene (LLDPE) was pre-irradiated by high energy electric beam under air atmosphere to produce peroxide on the LLDPE chains. Subsequently, the macromolecular free radicals produced by peroxide decomposition at high temperature reacted with MPS on SiO_2_-NPs surface during melt blending to form LLDPE-g-MPS-SiO_2_-NPs nanocomposites, as shown in Scheme 1. Besides, SiO_2_-NPs were modified by the inactive silane octyltrimethoxysilane (OTMS), forming OTMS modified SiO_2_-NPs (OTMS-SiO_2_-NPs), to prepare LLDPE/OTMS-SiO_2_-NPs nanocomposites for comparison. LLDPE/OTMS-SiO_2_-NPs nanocomposites have almost the same NPs dispersion but weak interface interaction comparing with LLDPE-g-MPS-SiO_2_-NPs nanocomposites. The results show that the LLDPE-g-MPS-SiO_2_-NPs nanocomposites displays superior DC breakdown strength to that fabricated by conventional modification methods.

## 2. Results and Discussion

### 2.1. SiO_2_-NPs Modification Results

**Figure 1:**
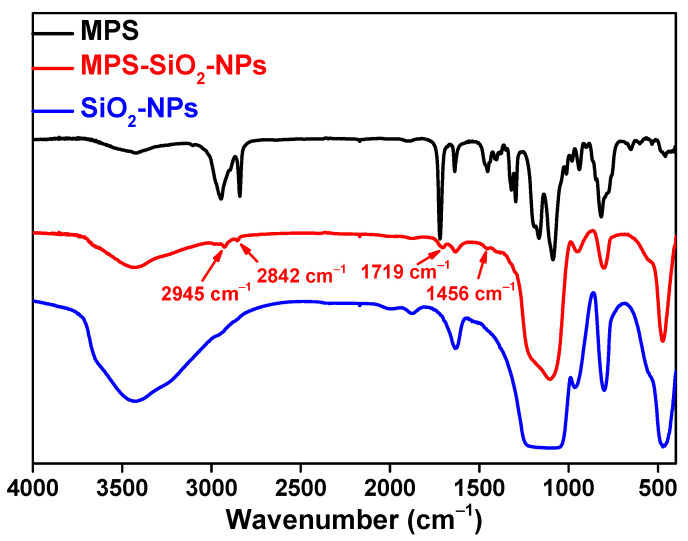
FT-IR spectra of MPS, MPS-SiO_2_-NPs and SiO_2_-NPs.

**Figure 2:**
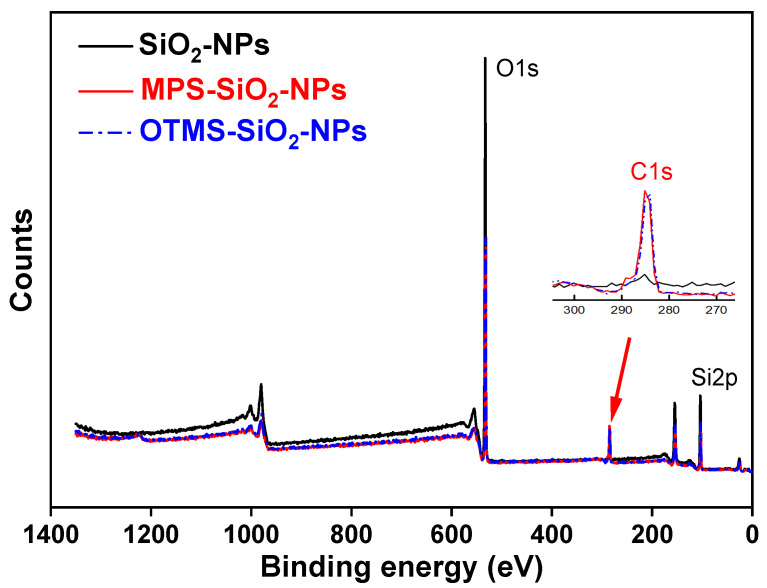
XPS spectra of pristine and modified SiO_2_-NPs.

Figure 1 shows the Fourier-transform infrared (FT-IR) spectra of the MPS, SiO_2_-NPs and MPS-SiO_2_-NPs. Compared to the pristine SiO_2_-NPs, several new peaks appear for the MPS-SiO_2_-NPs. The peaks at 2945 cm^−1^ and 2842 cm^−1^ represent the asymmetric and symmetric stretching vibration of -CH_3_, while the peak 1456 cm^−1^ can be ascribed to the bending vibration of -CH_3_. The peak at 1719 cm^−1^ can be assigned to the bending vibration of -C=O. All of these news peaks are corresponding to the characteristic peaks of MPS, suggesting that SiO_2_-NPs has been successfully modified by MPS. X-ray photoelectron spectroscopy (XPS) was used to analysis the composition of the SiO_2_-NPs and MPS-SiO_2_-NPs (Figure 2), and a new peak at 285 eV illustrates the presence of carbon on the MPS-SiO_2_-NPs surface (Figure 2 inset). Meanwhile, the peaks strength representing the O1s and Si2p in the MPS-SiO_2_-NPs spectra significantly decreases, demonstrating a successful grafting of MPS onto the surface of SiO_2_-NPs. Besides, it can be concluded from FT-IR spectra (Figure S1) and XPS spectra (Figure 2) that OTMS are also successfully covered onto SiO_2_-NPs surface.

**Figure 3:**
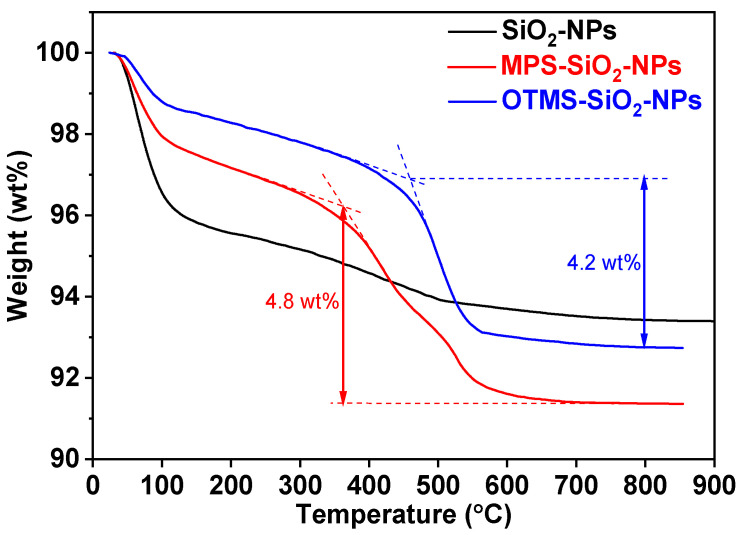
TGA curves of SiO_2_-NPs, MPS-SiO_2_-NPs and OTMS-SiO_2_-NPs.

The weight loss curves of various loadings of SiO_2_-NPs into the nanocomposites are shown in Figure 3. It can be seen that there is an obvious weight loss at ~100 °C on all thermogravimetric analysis (TGA) curves for the studied samples, which can be attributed to the loss of H_2_O adsorbed on the surface of SiO_2_-NPs. On one hand, the weight loss of SiO_2_-NPs is about 4%, which is almost 2 times higher than those of the MPS-SiO_2_-NPs and OTMS-SiO_2_-NPs. The presence of hydroxyl on the SiO_2_-NPs surface can easily absorb water from air. However, compared to the SiO_2_-NPs, the numbers of hydroxyl on the surface of MPS-SiO_2_-NPs and OTMS-SiO_2_-NPs significantly decreases (Figure 2), so the H_2_O absorption of NPs declines. On the other hand, the ester group of MPS shows hydrophilicity while the alkyl chain of OTMS shows hydrophobicity, so the OTMS-SiO_2_-NPs exhibits lowest weight loss around 100 °C. The second obvious weight loss for MPS-SiO_2_-NPs and OTMS-SiO_2_-NPs happens over 400 °C. Meanwhile, the initial decomposition temperature of OTMS-SiO_2_-NPs is higher than MPS-SiO_2_-NPs, which can be attributed to the decomposition of ester group on the MPS at low temperature. Considering different initial weight of water on the surface of the studied NPs, the content of the MPS grafted onto the MPS-SiO_2_-NPs is estimated over than 4.5 wt%.

### 2.2. Morphology and Structure of SiO_2_-NPs and LLDPE/SiO_2_-NPs Nanocomposites

**Figure 4:**
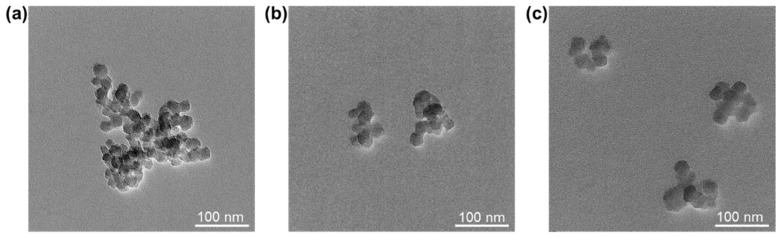
TEM images of (**a**) SiO_2_-NPs; (**b**) MPS-SiO_2_-NPs; and (**c**) OTMS-SiO_2_-NPs.

Transmission electron microscopy (TEM) images of all SiO_2_-NPs samples are shown in Figure 4. It can be seen that large aggregates appeared in the unmodified SiO_2_-NPs (Figure 4a). For MPS-SiO_2_-NPs or OTMS-SiO_2_-NPs, the size of aggregates become smaller (Figure 4b,c), suggesting superior dispersion. The results illustrate that the modification of silane coupling agent can inhibit the agglomeration between NPs. Besides, it should be noted that MPS-SiO_2_-NPs and OTMS-SiO_2_-NPs have almost the same dispersion level.

**Figure 5:**
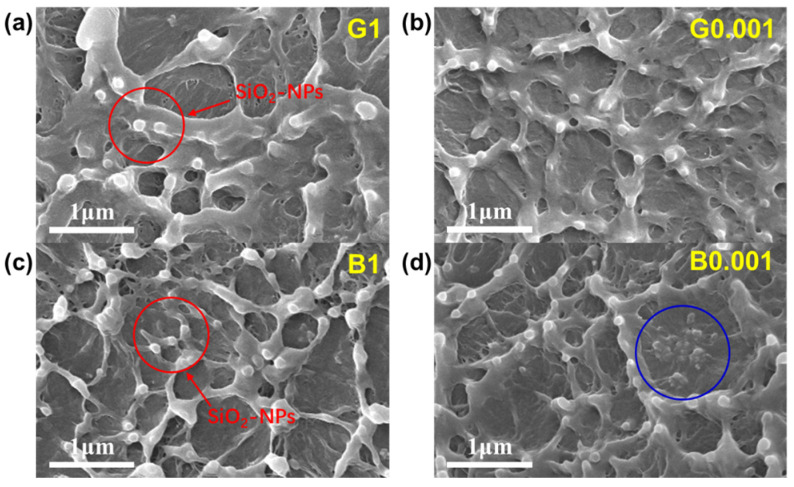
SEM images of LLDPE-g-MPS-SiO_2_-NPs (**a**) containing 1 wt% NPs and (**b**) 0.001 wt% NPs; and LLDPE/OTMS-SiO_2_-NPs (**c**) containing 1 wt% NPs and (**d**) 0.001 wt% NPs.

Scanning electron microscope (SEM) micrographs of G1, B1, G0.001 and B0.001 are shown in Figure 5. It can be observed that the SiO_2_-NPs are uniformly dispersed and embedded in the LLDPE highlighted by red circle in Figure 5a,c, whether the SiO_2_-NPs are grafted or just blended with LLDPE, implying that the dispersion of NPs improves after the surface was modified. After diluted with the LLDPE, the NPs exhibit the same dispersion (Figure S2). However, NPs aggregations can still be observed in the B0.001 samples (Figure 5d blue circle). The size of aggregations is about 0.5–1 μm, which is 10 times larger than the original size. These phenomena illustrated that the grafting strategies have a better effect on aggregation suppressing of SiO_2_-NPs than traditional surface modification methods.

### 2.3. Thermal Properties of LLDPE/SiO_2_-NPs Nanocomposites

**Figure 6:**
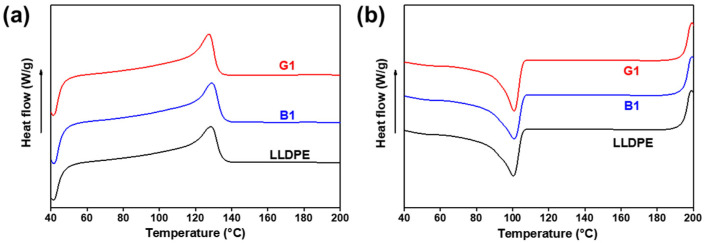
DSC (**a**) melting and (**b**) cooling curves of LLDPE and LLDPE/SiO_2_-NPs nanocomposites with 1 wt% of SiO_2_-NPs.

Figure 6 shows the differential scanning calorimetry (DSC) curves of the LLDPE, G1 and B1, and the key parameters are summarized in Table 1. It is found that the *T*_c_ and *T*_m_ of G1 and B1 are almost the same as those of neat LLDPE. However, the *X*_c_ of G1 and B1 decrease. G1 has a lower *X*_c_ compared with B1 because the addition of NPs can effectively strengthen the entanglement of polymer chains which inhibits the alignment of polymer chains to form crystal region [30]. Among these samples, the grafting of LLDPE onto the SiO_2_-NPs has the worst polymer chain mobility, resulting into a lowest *X*_c_ of G1. When the content of NPs in the nanocomposites is less than 0.5 wt%, the value of *X*_c_ slightly changes. But the LLDPE/OTMS-SiO_2_-NPs nanocomposites exhibit greater *T*_m_ and *T*_c_ dispersion (as shown in Figure S3 and Table S1). This may be due to a bad structural uniformity in LLDPE/OTMS-SiO_2_-NPs nanocomposites especially under ultra-low NPs loads.

### 2.4. Rheological Properties of LLDPE/SiO_2_-NPs Nanocomposites

**Figure 7:**
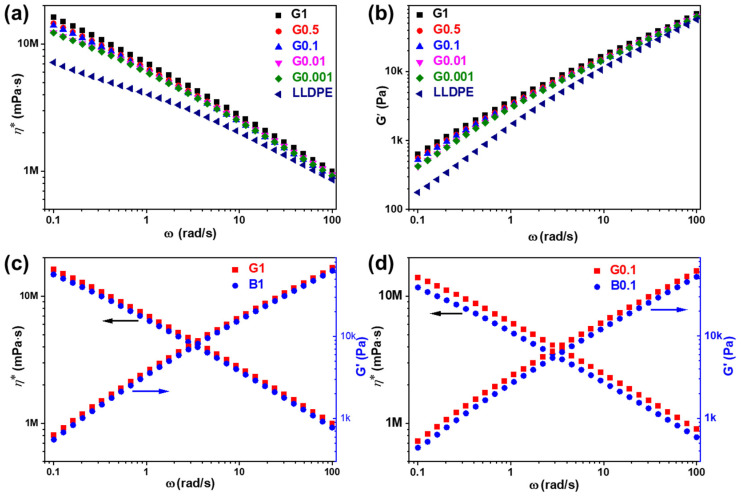
(**a**) complex viscosity and (**b**) storage modulus of LLDPE and LLDPE-g-MPS-SiO_2_-NPs; complex viscosity and storage modulus of nanocomposites with (**c**) 1 wt% and (**d**) 0.1 wt% SiO_2_-NPs modified with different methods.

The melt rheological properties of the LLDPE and LLDPE/SiO_2_-NPs nanocomposites were measured by rheology. Figure 7a,b and Figure S4a,b illustrate the frequency (*ω*) dependence of complex viscosity (η*) and storage modulus (*G*′) of the LLDPE and LLDPE/SiO_2_-NPs nanocomposites at 200 °C. LLDPE has a lowest *G*′ and η* corresponding to LLDPE-g-MPS-SiO_2_-NPs or LLDPE/OTMS-SiO_2_-NPs nanocomposites at all frequency ranges. When only 0.001 wt% of SiO_2_-NPs is added into LLDPE, the *G*′ and η* values for both LLDPE-g-MPS-SiO_2_-NPs and LLDPE/OTMS-SiO_2_-NPs nanocomposites have a large enhancement at a low frequency (0.1 rad/s). With further loading of SiO_2_-NPs into the nanocomposites, the values of *G*′ and η* slightly increase. These phenomena observed from the LLDPE/SiO_2_-NPs composites can be mainly ascribed to the formation of three-dimensional structure in polymer chains using SiO_2_-NPs as the bridge [31,32].

However, it is clearly showed that the *G*′ and η* of G1 is higher than B1 as shown in Figure 7c, confirmed by the observation when SiO_2_-NPs were diluted to 0.1 wt% in the nanocomposites (Figure 7d), indicating that MPS-SiO_2_-NPs significantly influenced the molecular relaxation than OTMS-SiO_2_-NPs. It should be noted that there is only weak interaction between LLDPE and SiO_2_-NPs (B1 and B0.1), because the OTMS on the SiO_2_-NPs surface has no chemical bond formation with LLDPE. Therefore, the LLDPE chains can slide against the OTMS-SiO_2_-NPs surface during rotary rheological test in a molten state. But in G1 and G0.1, the -C=C bonds of the MPS on the SiO_2_-NPs surface can easily chemically bond with the LLDPE to form LLDPE-g-MPS-SiO_2_-NPs nanocomposites at high temperature. Therefore, the strong linkage between LLDPE and MPS-SiO_2_-NPs are established. The LLDPE polymer chains that grafted onto SiO_2_-NPs surface can be easily entangled with other LLDPE chains in polymer matrix and form more physical crosslinks in the melt. As a result, the viscoelastic responses of the LLDPE-g-MPS-SiO_2_-NPs nanocomposites are enhanced compared to those of LLDPE/OTMS-SiO_2_-NPs nanocomposites. The η* and *G*′ of the LLDPE-g-MPS-SiO_2_-NPs and LLDPE/OTMS-SiO_2_-NPs nanocomposites with different SiO_2_-NPs concentration at 0.1 rad/s were summarized in Figure S4c,d, respectively. Both the η* and *G*′ for the LLDPE-g-MPS-SiO_2_-NPs nanocomposites are obviously higher than that of LLDPE/OTMS-SiO_2_-NPs nanocomposites in almost all of NPs composition ranges.

### 2.5. DC Breakdown Strength of LLDPE/SiO_2_-NPs Nanocomposites

**Figure 8:**
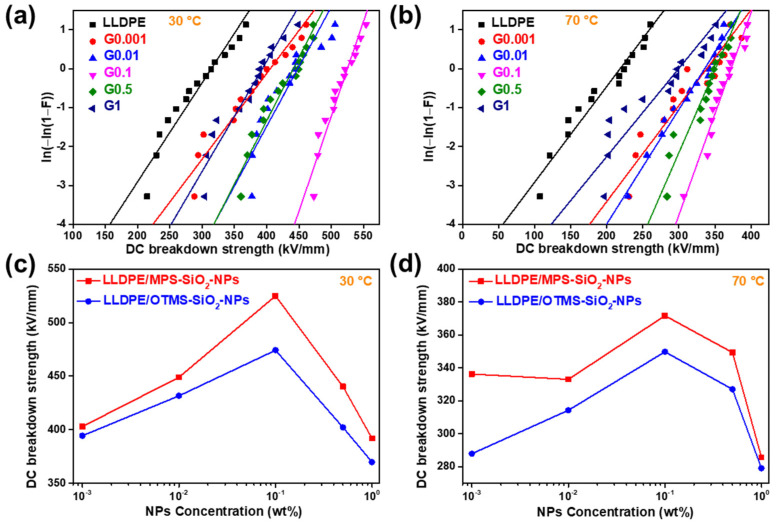
Weibull plots of DC breakdown strength of LLDPE and LLDPE-g-MPS-SiO_2_-NPs at (**a**) 30 °C and (**b**) 70 °C; the comparison of DC breakdown strength between LLDPE-g-MPS-SiO_2_-NPs and LLDPE/OTMS-SiO_2_-NPs at (**c**) 30 °C, and (**d**) 70 °C.

Dielectric strength is a critical property for insulating materials, because it determines the applied voltage and the life span of power instruments. The DC breakdown strength of all the samples were measured at 30 °C and 70 °C. The Weibull plots of DC breakdown strength of the LLDPE-g-MPS-SiO_2_-NPs nanocomposites with different concentrations of NPs are shown in Figure 8. The value of characteristic DC breakdown strength (*α*) and shape parameter (*β*) for the LLDPE-g-MPS-SiO_2_-NPs nanocomposites at 30 °C and 70 °C were summarized in Table 2 and Table 3, respectively. The DC breakdown strength of neat LLDPE is only 312 kV/mm at 30 °C. After doping only 0.001 wt% of MPS-SiO_2_-NPs into the LLDPE, the DC breakdown strength of LLDPE-g-MPS-SiO_2_-NPs nanocomposites can reach up to 402.9 kV/mm. When the content of MPS-SiO_2_-NPs further increases, the DC breakdown strength of LLDPE-g-MPS-SiO_2_-NPs nanocomposites first improves and then declines. The highest value of DC breakdown strength (525 kV/mm) is obtained at 30 °C for the sample G0.1, 69% higher than that of neat LLDPE. When the MPS-SiO_2_-NPs increases to 1 wt%, the DC breakdown strength reduces to 391.9 kV/mm, which is still approximately 25% higher than that of neat LLDPE.

Many theories are put forward in previous researches to explain the positive influence of NPs on dielectric properties [23,33,34]. It is widely accepted that the addition of NPs introduces large interface between inorganic NPs and polymer matrix, and brings more carrier traps. The high energy carriers injected from electrode can be captured by the traps to reduce their mobility, so the destruction of polymer chain could be reduced, making the improvement of puncture voltage of the insulating materials. However, the excess loading of NPs into polymer matrix not only introduces superfluous carrier traps but also severely generates defects, leading to two opposite effects: (i) superabundant carrier traps introduced by adding NPs in the polymer matrix significantly reduce the carrier mobility; (ii) the defects, such as voids, impurities, etc., seriously make dielectric failure.

When the dielectric strength was measured at 70 °C, the value of DC breakdown strength decreases. This may be attributed to higher carrier mobility at high temperature which could provide more opportunities for dielectric failure [35]. The DC breakdown strength of the LLDPE is only 214 kV/mm. After doping with MPS-SiO_2_-NPs, a high dielectric strength level can be achieved. The DC breakdown strengths of the studied nanocomposites are higher than 330 kV/mm at 30 °C, outperforming that of neat LLDPE under the same conditions. Meanwhile, the DC breakdown strength of LLDPE-g-MPS-SiO_2_-NPs nanocomposites is first increased and then decreases with the increasing of MPS-SiO_2_-NPs concentration. The highest value of 371.7 kV/mm at 70 °C is obtained which is 74% higher than neat LLDPE. Meanwhile, it could be found that the influence of NPs concentration on the DC breakdown strength at high temperature (70 °C) is reduced than that at low temperature (30 °C).

The value of sharp parameter (*β*) describes the dispersion of DC breakdown strength. The higher value of *β* represents a narrower data distribution, meaning that the material has a high reliability. But if the value of *β* is low, the reliability of the material is decreased. The neat LLDPE and G0.001 has almost the same *β* value which is lower than 10 at 30 °C. The *β* values of other studied nanocomposites are higher than 10, and the G0.1 has the highest *β* value of 23. These observations illustrate that the concentration of the NPs in the nanocomposites has a modulated effect on the performance uniformity, i.e., as the concentration of NPs is at low level, it is difficult for NPs to be evenly distributed throughout the spatial range of the polymer matrix, so the performance uniformity is destroyed. But when the concentration of NPs is at high level, more defects will be introduced into nanocomposites by NPs, and hence destroy the uniformity of microstructure. In the other word, the material reliability can be maintained at only a suitable NPs concentration. Besides, the *β* values of LLDPE-g-MPS-SiO_2_-NPs nanocomposites at 70 °C are much lower than that at 30 °C. This may be attributed to a reduction of structural consistency in amorphous region due to large segment relaxation ability of LLDPE polymer chains at high temperature considering that LLDPE is Semi-crystalline polymer.

The Weibull plots of DC breakdown strength of the LLDPE/OTMS-SiO_2_-NPs nanocomposites with different NPs concentration are shown in Figure S5, and the parameters are listed in Tables S1 and S2. It can be found that the DC breakdown strength of LLDPE/OTMS-SiO_2_-NPs nanocomposites is also higher than that of neat LLDPE at both 30 °C and 70 °C, meaning that traditional modified strategy of SiO_2_-NPs is helpful for enhancing the DC breakdown strength of nanocomposites. Besides, the results of DC breakdown strength of the LLDPE/OTMS-SiO_2_-NPs nanocomposites exhibits the same trend as that of the LLDPE-g-MPS-SiO_2_-NPs nanocomposites. But two obvious differences are observed: (i) the DC breakdown strength of the LLDPE-g-MPS-SiO_2_-NPs nanocomposites is about 10% higher than LLDPE/OTMS-SiO_2_-NPs nanocomposites under the same conditions within almost all range of NPs concentration at both 30 °C and 70 °C (Figure 8c,d); (ii) the *β* values of the LLDPE/OTMS-SiO_2_-NPs nanocomposites are much lower than that of the LLDPE-g-MPS-SiO_2_-NPs nanocomposites, suggesting that the uniformity of DC breakdown strength for LLDPE/OTMS-SiO_2_-NPs nanocomposites is worse than that of LLDPE-g-MPS-SiO_2_-NPs nanocomposites. Since the interface structure between NPs and polymer matrix plays an important role in the insulation properties. When the LLDPE is chemically linked at the surface of SiO_2_-NPs, the gaps between LLDPE and SiO_2_-NPs are sharply reduced, leading to an obvious reduction of the defects. Combined with DSC result that both *T*_m_ and *T*_c_ of the LLDPE/OTMS-SiO_2_-NPs nanocomposites have large data dispersion under low NPs loading, the LLDPE-g-MPS-SiO_2_-NPs nanocomposites have greater and more uniform DC breakdown strength than those of the LLDPE/OTMS-SiO_2_-NPs nanocomposites. These results verify that the successful grafting of LLDPE onto the SiO_2_-NPs during reactive extrusion can enhance the interface structure between SiO_2_-NPs and polymer matrix which will further increase the DC breakdown strength of nanocomposites.

## 3. Experimental

### 3.1. Materials

LLDPE (7042 powder) was obtained from Sinopec Maoming Company. SiO_2_-NPs (50 nm) were purchased from Macklin Co., Ltd. (Shanghai, China). MPS, OTMS and ammonium hydroxide were obtained from Aladdin (Shanghai, China). Anhydrous ethanol (EtOH) was obtained from Xilong Scientific Co., Ltd. (Shantou, China). The above reagents were used without any further purification.

### 3.2. Surface Modification of SiO_2_-NPs

Firstly, 3 g of SiO_2_-NPs were added in a 500 mL three-neck flask with 400 mL mixture of deionized water and EtOH (*v*/*v* = 1:9), and then ultrasonicated for 20 min. Subsequently, 1.8 g of silane coupling agent (MPS or OTMS) and a few drops of ammonium hydroxide were added and stirred by a magnetic stirring bar at 60 °C for 24 h. After the reaction, the modified SiO_2_-NPs were centrifuged, and cleaned by EtOH for three times to completely remove the unreacted silane coupling agent. The resulting products were dried at 60 °C for 24 h. The modified SiO_2_-NPs covered by MPS or OTMS were named as MPS-SiO_2_-NPs or OTMS-SiO_2_-NPs, respectively.

### 3.3. Preparation of LLDPE Grafting SiO_2_-NPs Nanocomposites

LLDPE was first pre-irradiated under air atmosphere with the total doses of 12 kGy at room temperature by a high energy electron beam accelerator. Then the pre-irradiated LLDPE and MPS-SiO_2_-NPs were mixed by a twin-screw extruder (SHJ-30, Giant, China) at a temperature setting of 130–200 °C to prepare LLDPE-g-MPS-SiO_2_-NPs nanocomposite. The modified SiO_2_-NPs were dried at 100 °C for 8 h under vacuum to completely remove H_2_O adsorbed on the surface of SiO_2_-NPs according to thermogravimetric analysis (TGA) results (Figure 3), and then the obtained nanocomposites were diluted by neat LLDPE to prepare samples with different contents of SiO_2_-NPs. The nanocomposites with 1, 0.5, 0.1, 0.01 and 0.001 weight percentages of SiO_2_-NPs were fabricated, abbreviated as G*n*, where *n* represents the content of nanoparticles in the as-fabricated samples. For comparison, the samples blending with OTMS-SiO_2_-NPs were also prepared via the same processing process and named as B*n*. Then the prepared nanocomposites were hot pressed under 10 MPa at 200 °C via a flat vulcanizer (Yangzhou, China) to prepare film samples for DC breakdown strength test.

### 3.4. Characterization of NPs Modification

All of the modified SiO_2_-NPs were performed by FT-IR spectra via a Nicolet IS10 spectrometer from 4000 cm^−1^ to 400 cm^−1^ with a resolution of 4 cm^−1^ in the transmittance mode. The XPS (K-Alpha+, Thermo Scientific, Waltham, MA, USA) was used to analyze the elements on SiO_2_-NPs surface. Both SiO_2_-NPs and the modified SiO_2_-NPs were investigated by TGA (DTG-60A, Shimadzu, Kyoto, Japan) with a heating rate of 10 °C/min from 40 °C to 900 °C under nitrogen atmosphere. The thermal behaviors of LLDPE and LLDPE/SiO_2_-NPs nanocomposites were recorded by DSC-7 (Perkin-Elmer, Waltham, MA, USA) in the temperature range from 40 °C to 200 °C with both heating and cooling rates of 20 °C/min. The crystallinity (*X*_c_) of LLDPE in the studied samples was calculated by following formula:

(1) $$X_{c}=\frac{\Delta H_{m}}{\Delta H_{m}^{\infty }}\times \frac{1}{\Phi \left(\mathrm{LLDPE}\right)}\times 100\%$$

where Δ*H*_m_ denotes the LLDPE melting enthalpy measured by DSC in every sample, ${\Delta H}_{m}^{\infty }$ represents the melting enthalpy of polyethylene with 100% crystalline and the value is 293 J/g [36], *Φ*(LLDPE) represents the weight fraction of LLDPE in the sample. The morphology of SiO_2_-NPs was identified using a TEM (HT-7700, Hitachi, Tokyo, Japan). The TEM characterization sample was fabricated by directly depositing the suspension containing SiO_2_-NPs onto an ultra-thin carbon film for observation. Fracture surface morphology of the samples was observed using a SEM (GeminiSEM 300, CARL ZEISS, Oberkochen, Batenwerburg, Germany). The fracture surface was coated with a thin gold layer before observation. The rheological properties of LLDPE and LLDPE/SiO_2_-NPs nanocomposites were studied by a rheometer (MCR102, Anton Paar, Graz, Austria) at 200 °C with the angular frequency of 10^−1^–10^2^ rad/s. The DC breakdown strength was tested at 30 °C and 70 °C on a GJW-50 kV voltage breakdown testing machine (Zhineng, Changchun, Jiling, China) with a ramp rate of 1 kV/s. To reduce the impact of impurities on the DC breakdown strength test, two small electrodes with a diameter of 6 mm were used and the film samples about 75 μm in thickness were sandwiched between them. The samples were immersed in silicone oil to avoid flashover during testing process. Two-parameter Weibull distribution was used to deal with the obtained DC breakdown strength data:

(2) $$F=1-exp\left[-{\left(\frac{E}{\alpha }\right)}^{\beta }\right]$$

where *F*, *E*, *α*, and *β* are the cumulative breakdown probability, the DC breakdown strength, 63.2% failure probability, and shape parameter, respectively. 15 LLDPE/SiO_2_-NPs samples were tested for an average value.

## 4. Conclusions

In this contribution, the linkage of MPS-SiO_2_-NPs and polymer matrix LLDPE can easily form by surface modifying the SiO_2_-NPs with MPS, followed by reaction with pre-irradiated LLDPE in the process of melt blending, which has many advantages for engineering application. FTIR and XPS results show that MPS is successfully coated on SiO_2_-NPs surfaces. SEM and TEM images illustrates that both distribution and dispersion of SiO_2_-NPs improve due to strong interface interaction between MPS-SiO_2_-NPs and LLDPE. LLDPE-g-MPS-SiO_2_-NPs nanocomposites exhibit approximately 10% higher DC breakdown strength than that of the LLDPE/OTMS-SiO_2_-NPs nanocomposites which has no obvious interaction between NPs and LLDPE, illustrating that conventional surface modification methods do not exploit the advantages of NPs as far as possible in improving material insulating properties. Therefore, this study may provide a new direction for investigating the effects of nanoparticles on electrical insulating. In addition, it is feasible that connecting other types of inorganic NPs directly to the polymer matrix to improve the insulation performances. Last but not least, polyolefin is one of the most important materials for industrial application, however it is difficult to be modified because of its inactive polymer chains. This research provides a chance to modify polyolefin with NPs to manufacture high-performance polyolefin nanocomposites, and the strategy is so simple that is perfectly suitable for engineering applications.

## Figures and Tables

**Table 1:** Summary of DSC data of LLDPE, G1 and B1.

Sample	*T*_m_ (°C)	*T*_c_ (°C)	Δ*H*_m_ (J/g)	*X*_c_ (%)
LLDPE	128.4	100.3	126.3	43.1
G1	127.4	100.6	98.6	33.6
B1	128.7	100.9	104.4	35.6

**Table 2:** DC breakdown strength of LLDPE-g-MPS-SiO_2_-NP_S_ at 30 °C.

Sample	*α* (kV/mm)	*β*
LLDPE	312	7.10
G0.001	403	8.32
G0.01	449	10.7
G0.1	525	23.7
G0.5	440	14.0
G1	392	10.2

**Table 3:** DC breakdown strength of LLDPE-g-MPS-SiO_2_-NP_S_ at 70 °C.

Sample	*α* (kV/mm)	*β*
LLDPE	214	4.89
G0.001	336	7.70
G0.01	333	10.4
G0.1	372	18.5
G0.5	349	15.7
G1	286	5.57

## Data Availability

The authors declare that all the data and plant materials will be available without restrictions.

## Supplementary Materials

The following supporting information can be downloaded at: https://www.mdpi.com/article/10.3390/molecules28134908/s1, Figure S1: FTIR spectra of OTMS, OTMS-SiO2-NPs and SiO2-NPs; Figure S2: SEM images of LLDPE-g-MPS-SiO2-NPs and LLDPE/OTMS-SiO2-NPs containing 0.5 wt%, 0.1 wt% and 0.001 wt% of SiO2-NPs; Figure S3: DSC cooling curves of (a) LLDPE-g-MPS-SiO2-NPs and (b) LLDPE/OTMS-SiO2-NPs; and DSC melting curves of (c) LLDPE-g-MPS-SiO2-NPs and (d) LLDPE/OTMS-SiO2-NPs; Figure S4: Rheological properties of (a) complex viscosity; (b) storage modules for LLDPE and LLDPE/OTMS-SiO2-NPs; (c) the complex viscosity and (d) storage modulus of all nanocomposites at 0.1 rad/s; Figure S5: DC breakdown Weibull plots of LLDPE/OTMS-SiO2-NPs: (a) at 30 °C and (b) at 70 °C; Table S1: Summary of DSC data of LLDPE-g-MPS-SiO2-NPs and LLDPE/OTMS-SiO2-NPs; Table S2: DC breakdown strength of LLDPE/OTMS-SiO2-NPS at 30 °C; Table S3: DC breakdown strength of LLDPE/OTMS-SiO_2_-NPS at 70 °C.

## Sample Availability

Samples of the compounds are not available from the authors.
