# Supplementary material for: The Improved DC Breakdown Strength Induced by Enhanced Interaction between SiO2 Nanoparticles and LLDPE Matrix
Source: Molecules. 2023 Jun 22;28(13):4908. doi: 10.3390/molecules28134908 (PMC10343384; doi:10.3390/molecules28134908)
Supplement: Supplementary file 1 [file molecules-28-04908-s001.zip › molecules-2444378-supplementary.pdf]

Supporting Information

The improved DC breakdown strength induced by enhanced interaction between SiO_2 nanoparticles and LLDPE matrix

Yaqing Lu, Yuyao Liu, Yujie Tong, Huili Cheng, Di Yang, Jiandong Ding, Qiyang Guo*

School of Chemistry and Chemical Engineering, Nantong University, Nantong 226019, People's Republic of China

*Correspondence Authors: qyguo@ntu.edu.cn

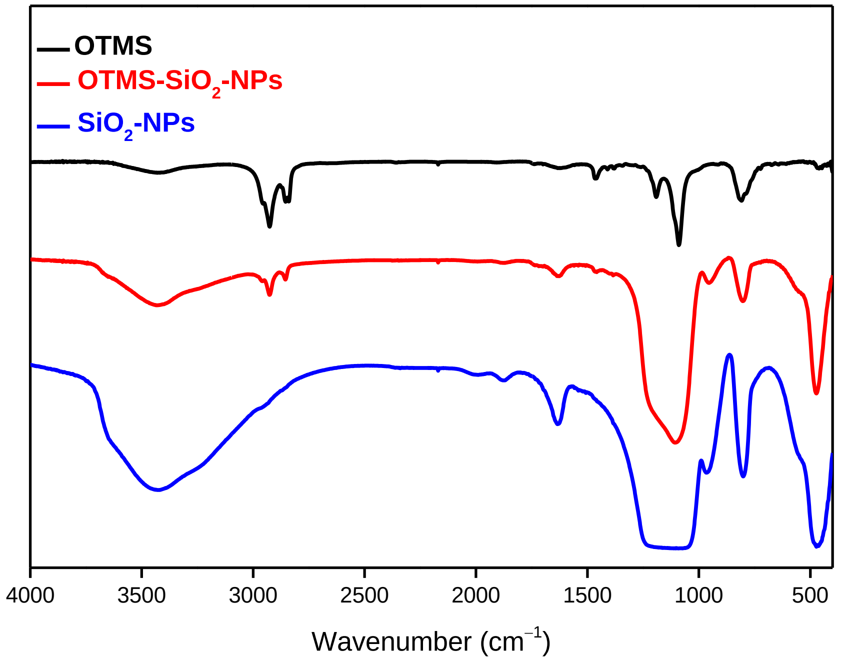

Figure S1. FTIR spectra of OTMS, OTMS-SiO_2-NPs and SiO_2-NPs.

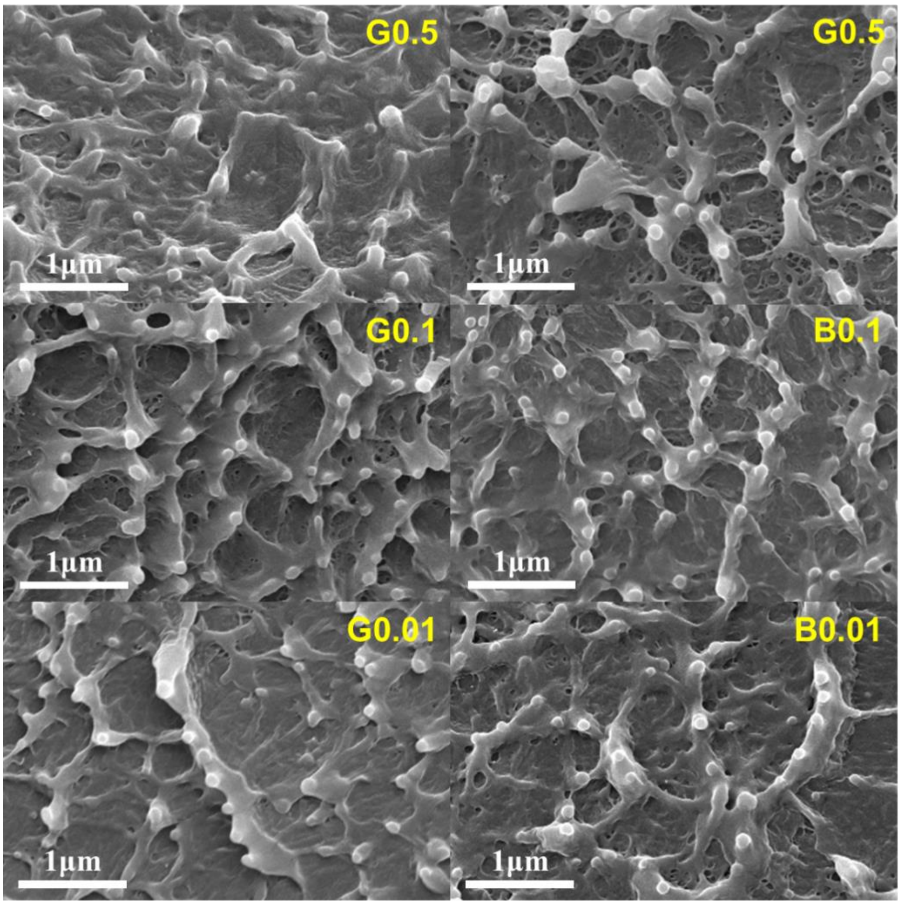

Figure S2. SEM images of LLDPE-g-MPS-SiO_2-NPs and LLDPE/OTMS-SiO_2-NPs containing 0.5 wt%, 0.1 wt% and 0.001 wt% of SiO_2-NPs.

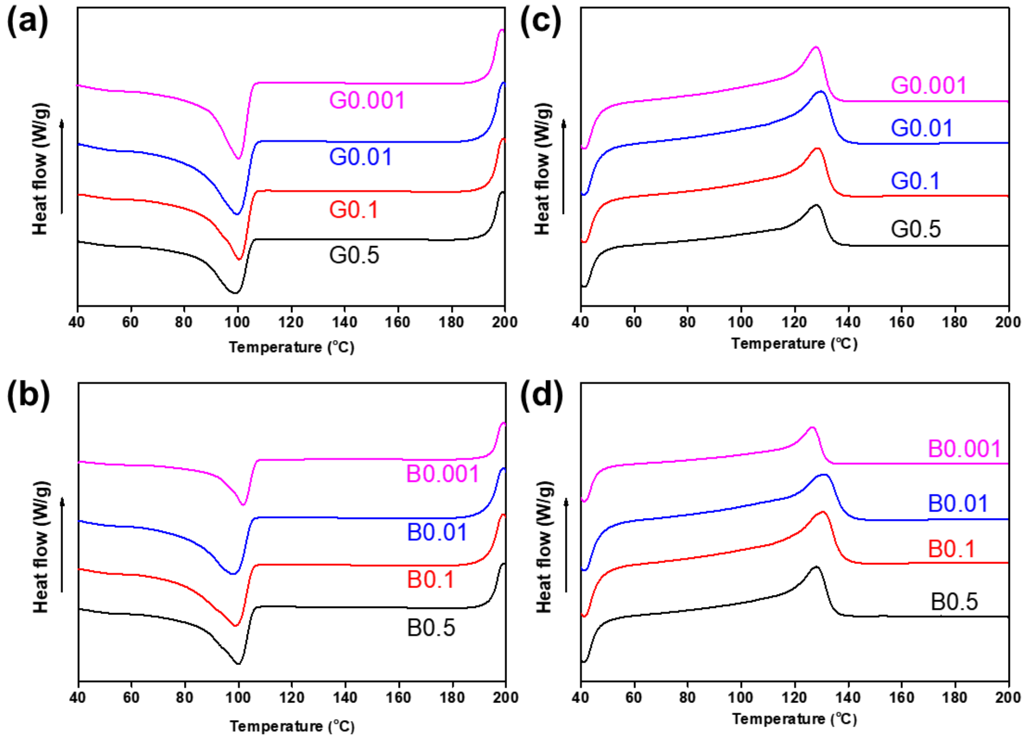

Figure S3. DSC cooling curves of (a) LLDPE-g-MPS-SiO_2-NPs and (b) LLDPE/OTMS-SiO_2-NPs; and DSC melting curves of (c) LLDPE-g-MPS-SiO_2-NPs and (d) LLDPE/OTMS-SiO_2-NPs.

Table S1. Summary of DSC data of LLDPE-g-MPS-SiO_2-NPs and LLDPE/OTMS-SiO_2-NPs.

Sample	T_m (°C)	T_c (°C)	ΔH_m (J/g)	X_C (%)
G0.5	128.0	98.9	106.0	36.2
G0.1	128.4	100.3	102.6	35.0
G0.01	129.7	99.3	103.1	35.2
G0.001	128.0	100.7	110.7	36.8
B0.5	128.0	99.9	105.6	36.0
B0.1	130.4	98.9	102.8	35.5
B0.01	130.7	97.9	105.5	36.0
B0.001	126.7	101.6	105.8	36.1

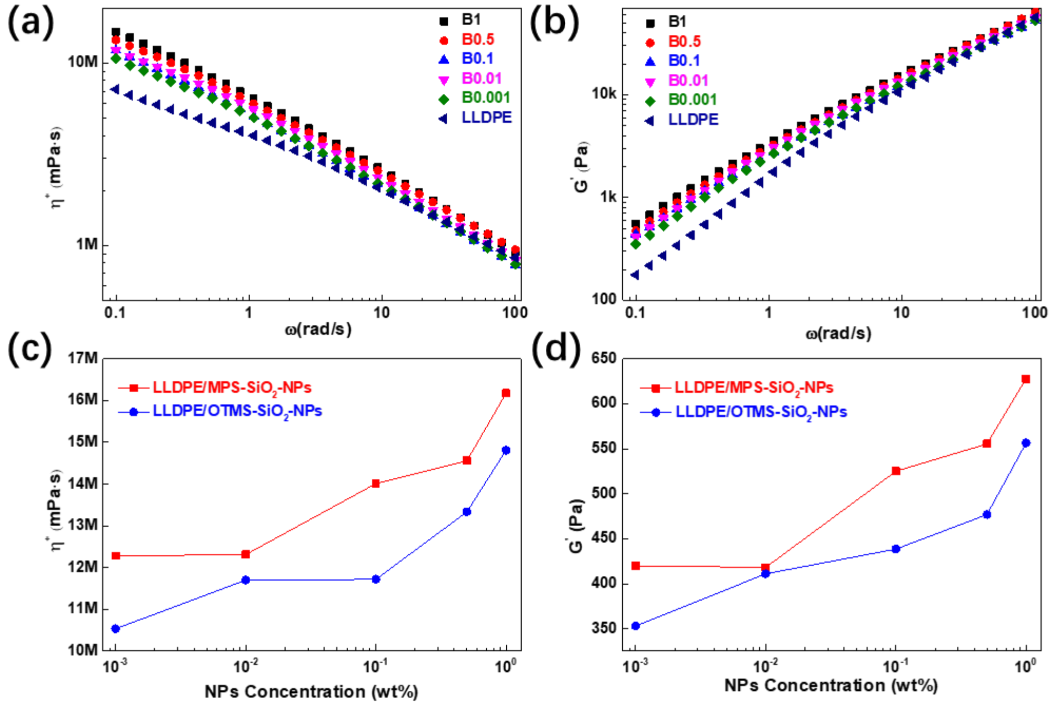

Figure S4. Rheological properties of (a) complex viscosity; (b) storage modules for LLDPE and LLDPE/OTMS-SiO_2-NPs; (c) the complex viscosity and (d) storage modulus of all nanocomposites at 0.1 rad/s.

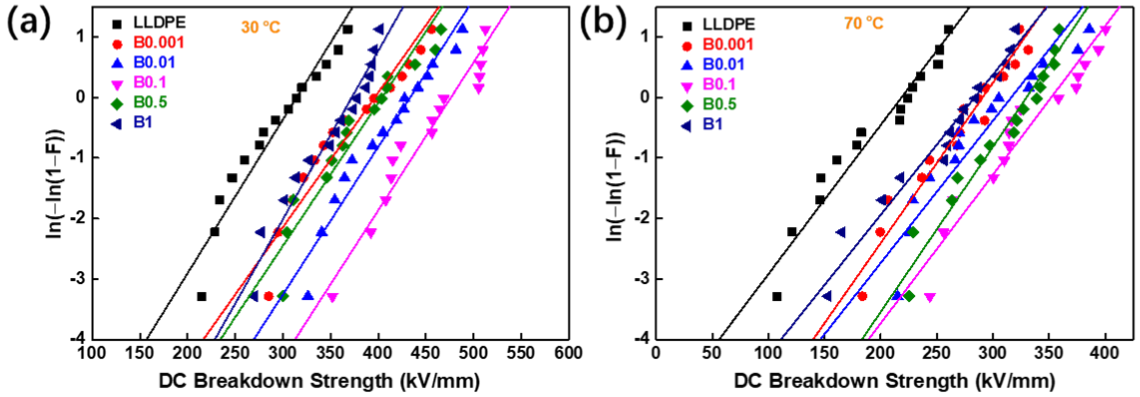

Figure S5. DC breakdown Weibull plots of LLDPE/OTMS-SiO_2-NPs: (a) at 30 °C and (b) at 70 °C.

Table S2. DC breakdown strength of LLDPE/OTMS-SiO_2-NPs at 30 ºC.

Sample	α (kV/mm)	β
PE	312	7.1
B0.001	394.3	7.83
B0.01	431.6	9.7
B0.1	474.3	11.35
B0.5	402.1	8.27
B1	369.8	10.92

Table S3. DC breakdown strength of LLDPE/OTMS-SiO_2-NPs at 70 ºC.

Sample	α (kV/mm)	β
PE	214	4.89
B0.001	287.9	7.43
B0.01	314.3	6.11
B0.1	349.8	7.88
B0.5	327.1	9.36
B1	279.0	6.66
